# Osteogenic Potential of Mesenchymal Stem Cells from Adipose Tissue, Bone Marrow and Hair Follicle Outer Root Sheath in a 3D Crosslinked Gelatin-Based Hydrogel

**DOI:** 10.3390/ijms22105404

**Published:** 2021-05-20

**Authors:** Hanluo Li, Hafiz Awais Nawaz, Federica Francesca Masieri, Sarah Vogel, Ute Hempel, Alexander K. Bartella, Rüdiger Zimmerer, Jan-Christoph Simon, Michaela Schulz-Siegmund, Michael Hacker, Bernd Lethaus, Vuk Savković

**Affiliations:** 1 Department of Cranial Maxillofacial Plastic Surgery, University Clinic Leipzig, 04103 Leipzig, Germany; Hanluo.Li@medizin.uni-leipzig.de (H.L.); Alexander.Bartella@medizin.uni-leipzig.de (A.K.B.); Ruediger.Zimmerer@medizin.uni-leipzig.de (R.Z.); Bernd.Lethaus@medizin.uni-leipzig.de (B.L.); 2 Institute of Pharmaceutical Sciences, University of Veterinary and Animal Sciences, Lahore 54000, Pakistan; awais@uvas.edu.pk; 3 School of (EAST) Engineering, Arts, Science & Technology, University of Suffolk, Ipswich IP 41QJ, UK; F.Masieri@uos.ac.uk; 4 Institute of Physiological Chemistry, Dresden University of Technology, 01307 Dresden, Germany; sarah.vogel@tu-dresden.de (S.V.); ute.hempel@tu-dresden.de (U.H.); 5 Clinic for Dermatology, Venereology and Allergology, University Hospital Leipzig, 04103 Leipzig, Germany; Jan-Christoph.Simon@medizin.uni-leipzig.de; 6 Institute for Pharmacy, University of Leipzig, 04103 Leipzig, Germany; schulz@rz.uni-leipzig.de; 7 Institute of Pharmaceutics and Biopharmaceutics, Heinrich-Heine-Universität Düsseldorf, 40225 Düsseldorf, Germany; michael.hacker@hhu.de

**Keywords:** hair follicle outer root sheath, mesenchymal stem cells, differentiation capacity towards osteogenic lineage, 3D injectable hydrogel, gelatin-based cross-linked hydrogel

## Abstract

Bone transplantation is regarded as the preferred therapy to treat a variety of bone defects. Autologous bone tissue is often lacking at the source, and the mesenchymal stem cells (MSCs) responsible for bone repair mechanisms are extracted by invasive procedures. This study explores the potential of autologous mesenchymal stem cells derived from the hair follicle outer root sheath (MSCORS). We demonstrated that MSCORS have a remarkable capacity to differentiate in vitro towards the osteogenic lineage. Indeed, when combined with a novel gelatin-based hydrogel called Osteogel, they provided additional osteoinductive cues in vitro that may pave the way for future application in bone regeneration. MSCORS were also compared to MSCs from adipose tissue (ADMSC) and bone marrow (BMMSC) in a 3D Osteogel model. We analyzed gel plasticity, cell phenotype, cell viability, and differentiation capacity towards the osteogenic lineage by measuring alkaline phosphatase (ALP) activity, calcium deposition, and specific gene expression. The novel injectable hydrogel filled an irregularly shaped lesion in a porcine wound model displaying high plasticity. MSCORS in Osteogel showed a higher osteo-commitment in terms of calcium deposition and expression dynamics of *OCN, BMP2*, and *PPARG* when compared to ADMSC and BMMSC, whilst displaying comparable cell viability and ALP activity. In conclusion, autologous MSCORS combined with our novel gelatin-based hydrogel displayed a high capacity for differentiation towards the osteogenic lineage and are acquired by non-invasive procedures, therefore qualifying as a suitable and expandable novel approach in the field of bone regeneration therapy.

## 1. Introduction

Bone is one of the most frequently transplanted tissues, accounting for over 2.2 million engraftments worldwide per year [1]. Bone autografts remain the simplest and most efficient application of bone graft; however, they are limited by their availability, size, and post-transplant donor site morbidity. The alternative, bone allografts available from donors are restricted by immunogenic responses from the host [2]. For these reasons bone regeneration based on tissue-engineered grafts, particularly those involving synthetic biocompatible materials have attracted immense attention in basic research, pre-clinical research, and translational applications. If optimal, a successfully engineered bone graft would mimic native bone morphological and functional characteristics, whilst integrating, repairing, and supporting innate regenerative processes. Three key elements make the optimal bone substitute: stem cells with high osteogenic capacity, an extracellular matrix (ECM) providing an osteo-inductive and osteo-conductive microenvironment with biomechanical support, and adequate exogenous differentiation cues.

Mesenchymal stem cells (MSC) have been extensively known as effective and promising cells of somatic origin for cell therapy and regenerative medicine, including engineered bone grafts [3,4,5]. MSCs are accessible from a multitude of tissues and can be derived both invasively, for example from human bone marrow [6] or adipose tissue [7,8] and non-invasively from umbilical cord blood [9] and hair follicle outer root sheath (ORS) [10].

MSCs display a particularly high potential for osteogenic differentiation that contributes to both osteogenesis and bone regeneration during development and postnatal bone repair. [11]. MSCs have an innate migratory ability, enabling rapid lesion site homing where they exert paracrine anti-inflammatory and trophic activity as well as take part in tissue replacement which is pivotal for tissue regeneration.

When a bone fracture occurs, MSCs play a key role in promoting differentiation of monocytes recruited at the lesion site towards the anti-inflammatory M2 macrophage via the NF-κB and STAT-3 signaling pathways [12]. In addition, they suppress the production of pro-inflammatory factors including TNF-α, IL-6, IL-12p70, and IFN-γ and enhance the secretion of anti-inflammatory cytokines such as IL-10 and IL-12p40 [13,14].

MSCs are successfully pre-differentiated in vitro using well-defined osteogenic supplements in a standard plastic-adherent culture [15]. However, in vitro 2D conditions drastically differ from the physiological in vivo environment, which often poses a barrier for effective osteogenesis in clinical applications. MSCs therefore require a suitable delivery tool for positioning them correctly within the lesion site of a bone defect whilst maintaining three-dimensional (3D) conditions resembling those of a physiological environment as closely as possible. The best strategy can be represented by a Combined Tissue Engineering Product (CTEP), providing an interface between a specifically fabricated artificial ECM (aECM) and appropriately selected stem cells. Ideally the aECMs designated for cell delivery should be non-toxic, biocompatible, biodegradable over time, and simple to apply. Equipping the aECM with the necessary flexibility for a delivery device and simultaneously mimicking the native extracellular matrix are the main challenges in engineering a graft, in particular for bone implants [16].

The commitment of MSCs to differentiate into specific cell lineages largely depends on the similarity of the aECM biochemical composition and biomechanics to those of the in vivo native environment [17]. MSCs display durotaxis due to their inherent pro-osteogenic function [18] and their adhesion, migration, and proliferation are significantly increased on stiff biomaterials [19]. This creates a paradox, as the ideal delivery device should simultaneously provide both the flexibility for filling irregularly shaped lesions and stiffness. This combination imposes additional demands on carriers for delivering stem cells and osteogenic precursor cells to the lesion site.

A delivery device made of collagen-containing hydrogel combined with cells may represent a valid experimental solution. Such a device would need to maintain its fluid phase for long enough to fill in lesions of any geometry in a custom-made fashion and it should also solidify quickly enough to maintain an even spread across the lesion. Such a graft would also need to remain permissive for diffusion of humoral cues. Hydrogel scaffolds composed of natural materials such as gelatin are very interesting for this purpose as they can successfully mimic the native ECM and provide flexibility. However, they lack the necessary stiffness. An approach to improve the stiffness of such natural hydrogels is represented by oligomer-crosslinking [20,21]. Hydrogels can also be enriched and functionalized by a cytokine load, in order to provide selected signals appropriate to the native cell niche and regulate proliferation, migration, differentiation, and apoptosis [22].

Gelatin is a naturally sourced degradation product of collagen, and it qualifies as an easily available source of biocompatible and biodegradable raw material for hydrogel fabrication. This allows for a wide choice of tissue engineering applications such as in skin repair and osteogenesis [23,24]. A serious limitation of gelatin-based hydrogels is a lack of stiffness due to thermal instability at physiological temperature, which is often solved by the cross-linking of gelatin chains. Cross-linking increases both mechanical and thermal stability, which also slows down in vivo degradation. Gelatin displays an abundance of aminoacidic residues, all potentially modifiable and particularly capable of reacting with a variety of cross-linkers, including the aforementioned oligomers [24].

In this study, we attempted to investigate the following: (1) testing the capacity for osteo-commitment of an autologous, non-invasively derived MSC from hair follicle outer root sheath (MSCORS) in a 3D differentiation environment; and (2) proof of concept for an appropriate adaptable delivery device combining MSCORS, BMMSCs, and ADMSCs with a gelatin-based functionalized hydrogel.

In order to address the two points above, we analyzed the combination of the MSCs with a previously reported injectable hydrogel for encapsulating living cells. This is obtained by mixing a hydrophilic pentaerythritol diacrylate monostearate (PEDAS)-based anhydride containing oligomer in a controlled reaction with a triethanolamine (TEOA) base allowing for an effective gelatin cross-linking [21]. The hydrogel used was selected from previously reported formulae available in a range of storage modules, which added to their extracellular matrix-like properties and could also be adjusted by the degree of cross-linking [25]. The low-molecular-weight quarter-oligomers used for the formulae varied in comonomer composition and contents of the reactive anhydride units, resulting in improved hydrogels hydrophilicity. This injectable hydrogel has previously demonstrated high cell compatibility and the capacity to support cell viability when combined with Human Adipose tissue-derived Stromal Cells (hASCs) [25].

Furthermore, this study aimed to compare the capacity for differentiation towards the osteogenic lineage of hydrogel-encapsulated MSCORS, with that of adipose tissue-derived MSCs (ADMSCs) and bone marrow-derived MSCs (BMMSCs), which also tested for the osteoconductive properties of the selected gel. A selection of MSCORS was particularly suitable compared to the ADMSCs and BMMSCs. Whilst ADMSCs and BMMSCs were invasively derived as per standard protocols, MSCORS were obtained in a scalable amount with a simple non-invasive procedure of hair plucking as previously described [10]. MSCORS are emerging as desirable MSCs not only for being readily available, autologous, non-invasive, and relatively easy to scale up, but also because they display a very good differentiation capacity towards the classic mesengenic lineages in vitro (adipogenic, chondrogenic, and osteogenic) as well as displaying potential towards endothelial- and smooth muscle lineages [10,26,27]. We compared capacity for differentiation towards the osteogenic lineage in vitro between the three MSCs (BMMSCs, ADMSCs, and MSCORS) in the selected gelatin-based oligomer-crosslinked hydrogel in 3D, which served both as a differentiation matrix as well as a delivery device with potential for future custom-made applications in bone regeneration.

## 2. Results

### 2.1. Isolation, Characterization, and Differentiation of MSCs

**Figure 1:**
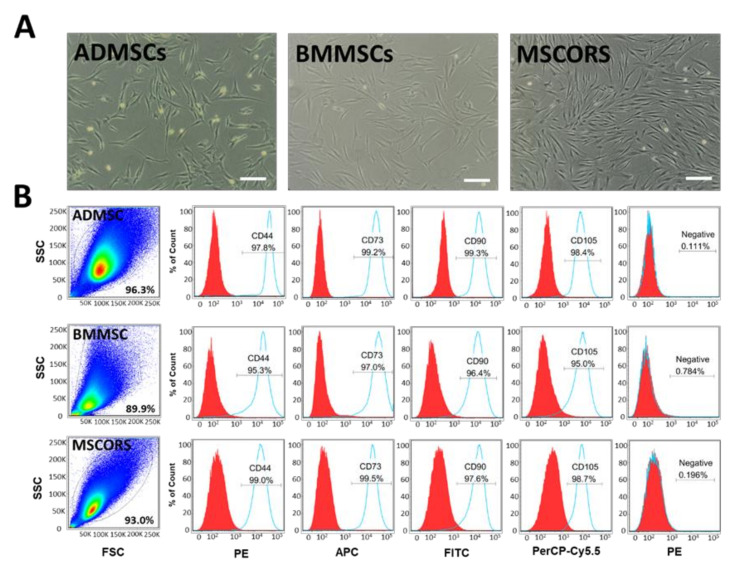
Phenotypic characterization and marker expression of adipose tissue-derived, bone marrow-derived and follicle outer root sheath-derived mesenchymal stem cells (MSCs) (ADMSCs, BMMSCs, and MSCORS). (**A**) Representative microphotographs of ADMSCs, BMMSCs, and MSCORS in culture at passage 2. (**B**) Representative histograms of the MSC marker expression levels against the isotype control in ADMSCs, BMMSCs, and MSCORS. FACS plots and histograms of representative labeling of ADMSCs, BMMSCs, and MSCORS with antibodies against human MSC surface markers CD44, CD73, CD90, CD105 (light blue bold curve) in comparison to labeling with the isotype control antibody (red filled curve). Negative markers included combination of the supplied negative MSC cocktail (PE CD45, PE CD34, PE CD11b, PE CD19, and PE HLA-DR). Scale bar: 200 μm; Magnification (**A**) 4×.

Adipose tissue-derived and bone marrow-derived mesenchymal stem cells (ADMSCs and BMMSCs, respectively) were used as controls for assessing the MSCORS culture encapsulation and differentiation capacity [10]. All three MSC types have shown cell culture plastic adherence capacity as expected and rapid proliferation in vitro *(*Figure 1A). Once stable primary cell cultures were obtained for the three cell types, the phenotype and marker protein expression were assessed.

MSCORS, ADMSC, and BMMSC from passage 2 to passage 4 were labeled with antibodies against MSC surface markers selected according to the International Society of Stem Cell Therapy (ISCT) criteria [28]. The cells were analyzed by flow cytometry, as shown in Figure 1B. The aggregated events in the obtained plots showed homogeneous cell populations in all the three MSC variants. MSCORS, ADMSC, and BMMSC expressed MSC-positive markers CD73, CD90, CD105, and CD44. All three cell types did not express MSC-negative markers CD45, CD34, CD11b, CD19, or HLA-DR, as shown in surface antigen analysis in Figure 1B.

The differentiation potential of MSCORS, ADMSC, and BMMSC including their differentiation towards osteogenic, chondrogenic and adipogenic lineages on a 2D surface were previously reported [10,29].

### 2.2. Fabrication of MSC-Encapsulated Osteogel and Immunostaining of CD90

**Figure 2:**
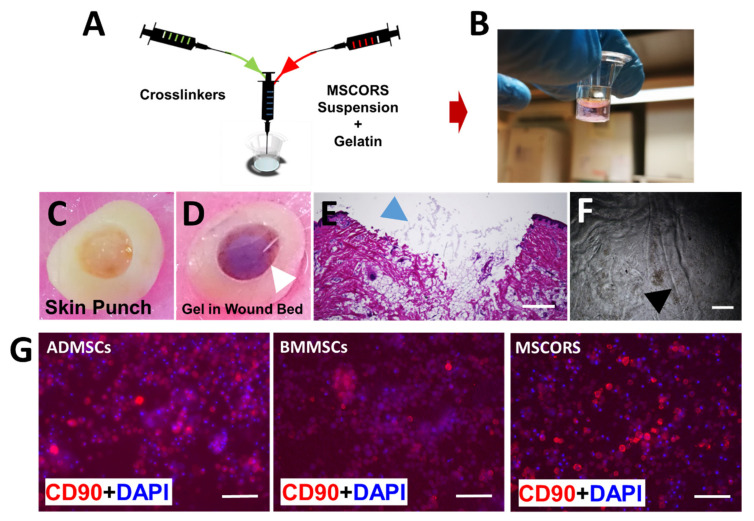
Osteogel production with MSCs encapsulation and semi-solid injectable Osteogel filling into an irregular wound bed of porcine skin model. (**A**) Production of cross-linked gelatin-based hydrogel and encapsulation of MSCs within the Osteogel. The cell suspension was first resuspended in a gelatin solution, and then co-injected together with the oligomer solution and triethanolamine (TEOA) base into a Transwell mold and mixed well. Gel formation was achieved by injecting the three components through an automatic pipette. The gelation process took place instantly and allowed for concomitant MSC encapsulation. (**B**) Osteogel in a Transwell mold. (**C**) Full-thickness punch lesion of a porcine skin was produced using a 5 mm biopsy punch. (**D**) Osteogel filled into the wound bed (white arrow). (**E**) H&E staining of the wound bed filled with the Osteogel (blue arrow), fitting completely the irregularly shaped lesion. (**F**) Crinkles pattern in the solidified hydrogel produced during stirring (black arrow). (**G**) Immunostaining of CD90 in ADMSCs, BMMSCs, and MSCORS grown for 7 days within the Osteogel. All MSCs displayed an intense CD90 cell surface signal, with MSCORS and ADMSCs being the most intense. Scale bar: €500 μm, (F) 250 μm, (G) 50 μm; Magnificatio€(E) 2×, (F) 4×, (G) 20×.

The gelatin-based cross-linked hydrogels were fabricated combining a gelatin solution with various oligomer solutions, as shown in Figure 2A,B. The gel composition and solution concentrations were previously reported [25].

To test the gelation capacity of the material, we employed a proof-of-concept model of a porcine ex vivo skin lesion. The lesion was obtained with a biopsy punch, where the different Osteogel components were co-injected with the cells resulting in their encapsulation. This allowed for an effective gelation process within the lesion, forming a smooth-sealed cover (Figure 2C). The hydrogel adhered to the irregular-shaped contours of the lesion and filled it before solidifying (Figure 2D,E). Histological cryosectioning of the embedded construct resulted in an artifact formation on the upper side of the gel, causing partial disruption to the continuity of the initially even Osteogel plug surface (Figure 2D,E).

To study cell phenotype within the Osteogel, immunostaining of the MSC biomarker CD90 was performed, with 4′, 6-diamidino-2-phenylindole (DAPI) nuclei counterstaining. BMMSCs, ADMSCs, and MSCORS were cultured for 7 days in a regular growth medium and displayed a spherical morphology within the Osteogel. The marker CD 90 showed a cell membrane-associated dotted distribution consistent with high expression of the marker, particularly in ADMSCS and MSCORS as shown in Figure 2G. A pattern of CD90 surface marker expression upkeep after 7 days in culture was similar to the one previously observed in 2D cell culture [10] and it was comparable between MSCORS, BMMSCs, and ADMSCs (Figure 2G).

**Figure 3:**
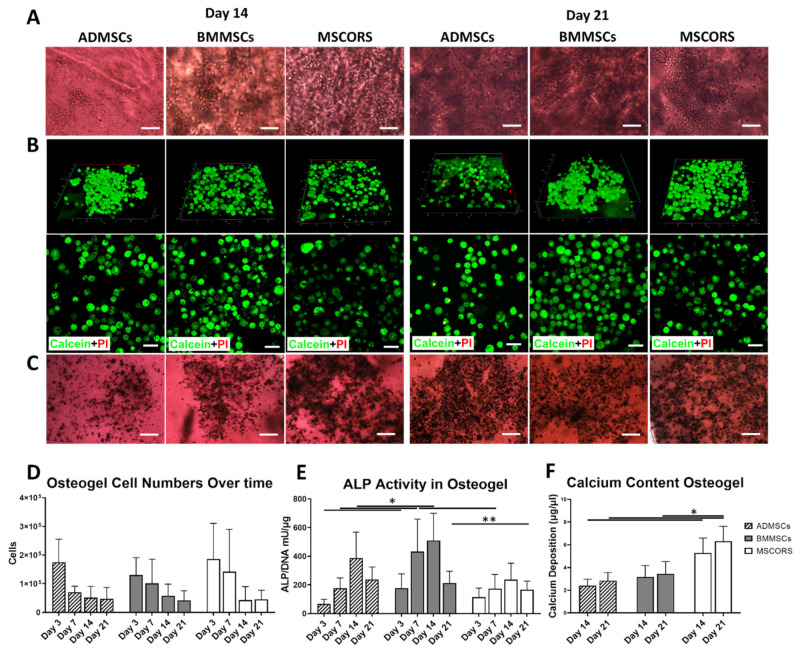
Osteogenic differentiation of ADMSCs, BMMSCs, and MSCORS in 3D in Osteogel. (**A**) Microphotographs of ADMSCs, BMMSCs, and MSCORS cultured in Osteogel under osteogenic differentiation conditions at day 14 and day 21. (**B**) Live/Dead assay of ADMSCs, BMMSCs, and MSCORS within the Osteogel, using Calcein AM (live/green) and Propidium Iodine (PI, dead/red), after osteogenic differentiation. (**C**) Alkaline Phosphatase (ALP) Visualization Assay based on 5-bromo-4-chloro-3-indolyl phosphate/p-nitroblue tetrazolium chloride (BCIP/NBT) chromogenic reaction in ADMSCs, BMMSCs, and MSCORS after Osteogel-based 3D osteogenic differentiation. (**D**) Cell numbers over the differentiation period quantified by DNA Picogreen assay at day 3, 7, 14, and 21. (**E**) Quantitative analysis of ALP activity vs. DNA content. ALP activity in MSCs was assessed using pNPP chromogenic assay over day 3, 7, 14, and 21 of differentiation. (**F**) Calcium deposition in ADMSCs, BMMSCs, and MSCORS quantified by the CPC Assay. Data are shown as mean ± SD (* *p* < 0.05). Comparison of each group was statistically analyzed by unpaired Student’s t-test or nonparametric Mann–Whitney test. Statistical significance: * *p* < 0.05, ** *p* < 0.01. Scale bar and magnification: (**A**) 100 μm, 20×; (**C**) 200 μm, 10×; (**B**) 50 μm, 63×.

All MSCs exhibited a spherical morphology after encapsulation in the gelatin-based crosslinked Osteogel as shown in Figure 3B, accounting for accurate 3D-environment cell embedding. The gel solidification pattern followed the stirring direction during the hydrogel production, as documented by microscopic imaging (Figure 2F). During the process of rapid crosslinking and quick mixing by stirring, non-homogeneous gelation crinkles were formed resulting in a line-like pattern and intensive refraction accounting for artifacts. Notably, these did not affect the cell positioning.

### 2.3. Osteogenic Differentiation of MSCs in 3D-Osteogel

ADMSC, BMMSC, and MSCORS underwent differentiation under osteogenic conditions in a 3D environment at day 14 and day 21 (Figure 3A). After 21 days of growth in osteo-inductive conditions, MSCs displayed a rounded shape morphology with good refraction as captured by bright field microscopy. The cell monolayer was covered with an opaque layer of extracellular CaP mineral deposit.

A Live/Dead Assay with Calcium AM (green fluorescence) and Propidium Iodide (PI) (red fluorescence) was used to assess MSC cell viability in the Osteogel during 3D differentiation in osteogenic medium. Reconstructed confocal z-stacking images of the 3D-cultured cells stained with Live/Dead assay are shown in Figure 3B. The majority of the cell population showed an intense Calcein signal with clear dotted distribution throughout the cytoplasm, indicating high cell metabolism and viability. The in silico 3D reconstruction of fluorescence signals highlighted that upon differentiation in osteogenic conditions the cells acquired a 3D spherical morphology and an even distribution accounting also for an initial even distribution of the MSCs. Virtually undetectable levels of cell apoptosis were recorded by red PI staining (Figure 3B). Some low-intensity signal arising from the anisotropic hydrogel due to a likely phenomenon of Calcein fluorescent signal scattering refraction was also documented and considered as an artifact (Figure 3B).

To determine the activity of ALP in the ADMSC, BMMSC, and MSCORS after 14 and 21 days of growth in osteogenic medium an ALP assay was used as shown in Figure 3C. The differentiated MSCs visible within the Osteogel were stained intensively dark brown, indicating that all three MSC types had a high ALP content.

The number of living cells decreased during differentiation towards osteogenic lineage in all the three cell types, MSCORS, BMMSCs, and ADMSCs (Figure 3D).

To further assess the overall ALP activity, a quantitative ALP assay was used as displayed in Figure 3E. ALP quantitative analysis showed the same modulation trends in all three MSC types, with an activity increase from day 3, a peak on day 14, and a subsequent decrease by day 21 as shown in Figure 3D. BMMSC displayed higher ALP activity compared to that of ADMSCs and MSCORS (by 1.4-fold to ADMSC and 1.5-fold to MSCORS) in particular from day 7 to day 14 (*p* < 0.05).

**Figure 4:**
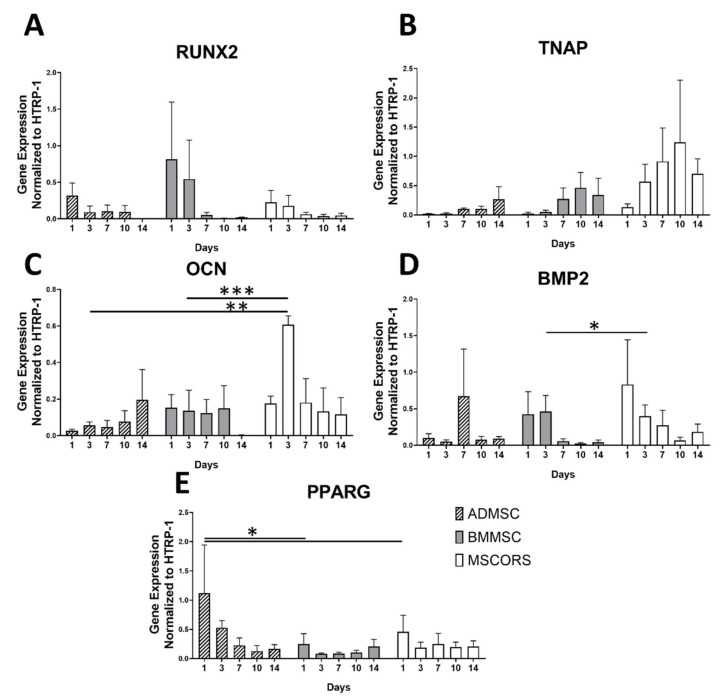
Gene expressions of osteogenic and adipogenic markers analyzed with qRT-PCR in ADMSCs, BMMSCs, and MSCORS during 3D differentiation in Osteogel, at day 1, 3, 7, 10, and 14 of differentiation. The analysis includes *RUNX2* (**A**), *TNAP* (**B**), *OCN* (**C**), and *BMP2* (**D**), and adipogenic gene *PPARG* (**E**). Relative expression levels were normalized to the housekeeping gene *HPRT-1* and shown as mean ± SEM. Comparison of each group was statistically analyzed by the unpaired Student’s t-test or nonparametric Mann–Whitney test. Statistical significance: * *p* < 0.05, ** *p* < 0.01, *** *p* < 0.001.

The deposition of calcium phosphate (CaP) was assessed by CPC Assay, to quantify the functionality of osteoblasts-like cells differentiated from ADMSCs, BMMSCs, and MSCORS in the Osteogel (Figure 3F). Notably, the deposition of CaP seen in MSCORS at both day 14 and day 21 time points was significantly higher than that of ADMSCs (day14: 2.19- fold, *** *p* = 3.11 × 10^−8^; day 21: 2.22- fold, *** *p* = 2.03 × 10^−9^) as well as higher than that measured in BMMSCs at day 21 (1.83- fold, *** *p* = 6.53 × 10^−7^). The observed CaP deposition patterns were in line with *TNAP* and *OCN* gene expression levels in MSCORS (Figure 4B,C).

### 2.4. Osteogenic Gene Expression in 3D-Osteogel Differentiation

Gene expression levels of osteogenic markers *RUNX2, TNAP, OCN, BSPII, BMP2*, and the adipogenic marker *PPARG* during 3D differentiation of the three MSC types grown in Osteogel were assessed by qRT-PCR (Figure 4). Relative gene expressions revealed varying levels of osteogenic marker expression in ADMSCs, BMMSCs, and MSCORS, with consistent trend expression dynamics throughout the differentiation process in Osteogel.

In ADMSCs, BMMSCs and MSCORS, *RUNX2* was highly expressed at day 1, and declined during the 21-day differentiation period. BMMSCs showed higher yet not significant expression levels of *RUNX2* particularly at day 1 and day 3, compared to the other cells. Expression dropped from day 7 and remained very low until day 21. ADMSCs and MSCORS had an overall lower expression level of *RUNX2* in comparison to BMMSCs; however, the expression level was higher at day 1 similarly to what was observed for BMMSCs. Similar non-significant trends were also observed for *TNAP* expression in all tested MSCs, which gradually increased from day 1, peaked at day 14 in ADMSCs, and at day 10 in BMMSCs and MSCORS, respectively.

In contrast, expressions of *OCN* and *BMP2* varied between ADMSCs, BMMSCs, and MSCORS. In ADMSCs *OCN* expression increased gradually from day 1 and peaked on day 14. In BMMSCs *OCN* expression remained steady and decreased on day 14. The expression of *OCN* in MSCORS increased on day 3 and declined slowly from day 7 onwards. The peak expression of *OCN* in MSCORS at day 3 was significantly higher than that of ADMSCs (*** *p* = 0.00024) and BMMSCs (** *p* = 0.00946) (Figure 4C). *BMP2* expression displayed a similar decreasing trend in both BMMSCs and MSCORS differentiation, whereas in ADMSC it peaked on day 7 and subsequently declined until day 14. OCN expression level in MSCORS was higher than that of in BMMSC by 315.35% on day 14 (* *p* = 0.04019).

ADMSCs showed the highest expression level of the adipogenic marker *PPARG* on day 1 and declined from day 2 (ADMSCs to BMMSCs by 549.69% *p* = 0.012 < 0.05, ADMSC to MSCORS by 185.56% *p* = 0.0485 < 0.05, Figure 4E). *PPARG* expression in BMMSCs and MSCORS was comparatively low, showing the highest expression level on day 1, followed by a steady decline until day 14.

## 3. Discussion

In this study, MSCORS were embedded in a biocompatible in situ gellable matrix, providing a versatile platform to engineer the co-injection of a mix of cells with an adaptable material. This may serve for future proof of concept work towards minimally invasive, personalized approaches to bone regeneration treatment that are easily scalable in vivo and transferable to the clinical setting. Moreover, by comparing MSCORS behavior within the engineered hydrogel to that of ADMSCs and BBMSCs we have categorized them as a feasible component of an optimal CTEP.

Upon a non-invasive plucked hair follicles and outgrowth method, MSCORS fulfilled the characterization profile of MSCs [10]. MSCORS demonstrated MSC-like dendritic morphology and rapid proliferation in vitro, adherence to plastic, forming of colonies, and a potential to differentiate into mesodermal tri-lineages including osteoblasts, chondrocytes, and adipocytes, as previously reported [8,10]. MSCORS, along with ADMSCs and BMMSCs expressed MSC-positive surface markers CD73, CD90, and CD105 and did not express any of the MSC-negative surface markers CD34, CD45, CD14, or CD11b, CD79α or CD19, and HLA-DR [7,28,30,31,32,33,34] as defined by the ISCT MSC characterization criteria and demonstrated by flow cytometric analysis (Figure 1B). MSCORS showed comparable expression levels of MSC positive markers with ADMSCs and BMMSCs which have been considered to be the MSC golden standard in past three decades [35]. Notably the expression profile of MSC-positive markers was persistently high and more stable during culture in MSCORS than in ADMSCs, regardless of inter-individual donor differences. These results are in accordance with previous reports of ADMSCs [7,8], BMMSCs [32], and MSCORS [8] expressing immunologically relevant cell surface markers [28].

The 3D culture Osteogel system presented an adaptable gelatin-based cell-aECM interface and it supported an effective differentiation of the ADMSCs, BMMSCs, and MSCORS towards the osteogenic lineage. Cell viability, expressions of osteogenic genes, ALP activity, and CaP production were investigated at different timepoints in a 3D environment model. All MSCs showed spherical morphology, high viability, retained optimal MSC-relevant phenotypic features, and differentiated into functional osteoblast-like cells with a high degree of ALP activity and CaP deposition in the gel (Figure 3A–F). The observed ALP activity in the Osteogel corresponded with previous findings in human bone marrow MSC osteogenic differentiation in 2D, which displayed a peak of ALP activity on day 14 [36]. The differences in ALP activity between the three MSC types remained at a comparable to non-significant trend level. This may be due in part to the large variability between donors. The CaP depositions in Figure 3E indicate that MSCORS in the Osteogel produced significantly higher amount of CaP than that measured in ADMSCs and BMMSCs grown in the same conditions (Figure 3C). MSCORS and ADMSCs showed comparable levels of ALP activity, which is used as an intermediate criterion of osteo-differentiation. Those levels were slightly lower to comparable to those of BMMSCs. This can be used as a criterion of successful osteo-differentiative performance in a 3D in vitro environment and suggests a similar efficiency of the differentiative process in its mid-phase in all three tested MSC types. When considering CaP deposition as an endpoint criterion for assessing the differentiation towards the osteogenic lineage, MSCORS performed better compared to BMMSCs and ADMSCs. This feature of MSCORS exhibited in Osteogel may be of major significance and a main advantage for their future application potential in supporting osteoinductive protocols.

Gene expression analysis at multiple time points during osteogenic induction provided an insight into the process of MSC differentiation capacity towards the osteogenic lineage. Osteogel-encapsulated cells were induced by using a standard osteogenic medium containing Dexamethasone (Dex), Ascorbic Acid (Asc), and β-glycerophosphate (β-Gly).

The Osteogel used consisted of a matrix rich in type I collagen. It has been known that interactions between type I collagen from the ECM and α2β1 Integrin from the MSCs/Osteoblasts activate the MAPK signaling leading to the phosphorylation and activation of Runx2, which initiates transcription of the osteoblast marker genes [37]. Accordingly, we measured the expression of *RUNX2*, *TNAP*, *OCN*, and *BMP2* genes.

The osteogenic medium, together with the osteo-conductive properties of the gel lead to an increase in expression of *RUNX2* in line with previously observed Ihh-, MAPK-, and Wnt/β- related gene expression pattern studies in 2D osteogenic differentiation [38]. Moreover, a decrease of *PPARG* expression and the increase of *OCN* expression (Figure 4) had been previously reported during the osteogenic differentiation of BMMSCs, which may confirm the trends in our findings [39].

In this study, we noted similar expression dynamics of *TNAP* expression (Figure 4), in line with a previously reported increase in expression of *OCN* and ALP in human BMMSCs in hypoxic culture settings for 7 days [40], reaching its peak at day 14 and subsequently decreasing towards day 21 [41].

The ECM plays a pivotal role in maintaining the cellular microenvironment integrity whilst providing cues that control differentiation, in particular type I collagen is crucial for MSC osteogenic commitment and differentiation [42]. The Osteogel aECM rich in denatured type I collagen cross-linked using anhydride-containing oligomers, mimicked well the principal architecture of the native ECM contributing to support the differentiation of MSCORS, ADMSCs, and BMMSCs towards the osteogenic lineage in a 3D in vitro setting. This provides a potential interface for biochemical signals elicited by the components of the osteogenic medium, opening a rationale for future applications. Osteogel was sufficiently supportive in 3D differentiation towards the osteogenic lineage, without a need for additional functionalization by load-and-release of further signaling molecules; this may be a subject of future optimization efforts.

The mechanical properties of aECMs exert a clear impact on stem cell behavior and osteogenic differentiation, mediated by mechano-transduction [42]. In this process, the osteogenic signals are primarily transduced by integrin-linked kinase and focal adhesion. In MSCs, those signals are dependent on *Vinculin* expression, ECM-integrin interaction, and YAP/TAZ activation via Ras superfamily Rho GTPases and Rho/ROCK signaling [43,44,45]. Rho/ROCK signaling was also reported to dominantly regulate osteogenic differentiation of periodontal ligament cells, displaying corresponding *RUNX2* and ALP expression levels to those observed in bone marrow-derived MSC [45]. *RUNX2* expression in BMMSCs was found positively correlated to the matrix stiffness and the surface ligand availability. A previously reported study employed a low-stiffness matrix of 0.7 kPa storage modulus, which reduced *RUNX2* expression and the subsequent osteogenic differentiation of BMMSCs grown on said matrix [42]. The Osteogel used in this study is a relatively soft hydrogel with a storage modulus up to 0.5 kPa upon gelation, and 2.75 kPa after day 1 of exposure to cell culture conditions [25]. Here, we reported how Osteogel-interface with cells impacted differentiation towards the osteogenic lineage in osteoinductive conditions, reflected by the variable osteogenic gene expression levels. As shown in Figure 4A, the dynamics of *RUNX2* expression in the 3D Osteogel were consistent with the findings of Sun et al. [46] during MSC osteogenic differentiation in similar 3D hydrogel systems with varying stiffness analyzed in mice [46]. In our study, *RUNX2* expression was particularly high on day 1 of differentiation in all three tested MSC types and from then on it gradually declined until day 14 as shown in Figure 4A. Additionally, the expression levels of *TNAP* and *OCN* assessed in this study correspond to the *TNAP* and *OCN* expression dynamics reported by Sun et al., which showed a consistent expression rate increase until day 7 correlating negatively with the matrix stiffness [46]. Relying on the moderate stiffness of the Osteogel used here and on the reported conclusions of the mentioned study, we argue that Osteogel might have prompted an earlier onset expression of *RUNX2* and other related genes including *OCN*, especially when compared to other well-established relatively low-stiffness materials such as polyacrylamide hydrogel [46].

The cells encapsulated in the Osteogel showed high survival rates. We attempted to interpret the possible reasons for the remarkably low residual cytotoxicity observed. The spherical morphology of the cells was easily maintained in the Osteogel however the semi-solid build may also account for partially arresting cell proliferation, just as any other semi-solid aECM [47,48]. The organic solvents used for oligomer crosslinking, Dimethyl sulfoxide (DMSO) and N-Methyl-2-pyrrolidone (NMP) could have also exerted a certain degree of cytotoxicity in 3D hydrogel that provides an all-around contact surface [47,48]. For these reasons, the hydrogel formulation that contained TEOA was selected as a solvent known to be safe for cells and it was applied in an amount below toxicity levels in the final Osteogel preparation. The cytotoxic potential of DMSO and TEOA has been previously discussed in the context of cell proliferation and did not appear to cause cell death in the injectable hydrogel, despite the stress caused to the cells by previous trypsinization and subsequent encapsulation processes [25]. The same report describes the challenges of determining cell numbers within the hydrogel using a biochemical WST-1 assay, which may be hindered by the intrinsic absorbing properties of the gel. To obtain accurate results we determined the number of cells according to their DNA content. The decline in cell number that occurred during osteogenic differentiation was expected for differentiating cells and it could have been additionally caused by spatial limits imposed by mineral deposition as the differentiation progressed. MSCORS maintained high numbers of viable cells during early osteo-commitment, as shown in Figure 3D. The higher number of viable cells was likely responsible for depositing higher amounts of CaP (Figure 3F) which illustrates a favorable cumulative output of the optimized MSCORS-based experimental set-up.

The 3D differentiation in the Osteogel supported a better defined osteo-progenitor cell morphology when compared to that of cells undergoing 2D osteo-differentiation reported in [10]. It can be argued that the spatial distribution comes as an innate advantage of a 3D cell culture setting, which also allows for a more physiological mineralization process by resembling the in vivo bone geometry more closely.

The key feature addressed in this study was the ability of MSCs, in particular MSCORS, to favorably respond to osteo inductive cues in the Osteogel and produce differentiated pre-osteoblast precursors that resulted in an osteoblast-like phenotype and function. By virtue of quick gel formation, the Osteogel qualifies as an efficient delivery device since it can be applied to inaccessible lesions extremely quickly. Once gelled, the Osteogel maintains the 3D- encapsulated cells spreading at an equal distance (Figure 3B), which eliminates clumping. As such, the Osteogel could also be used to deliver the pre-osteoblasts to a lesion site by minimally invasive local injection and adjust its final form to the site geometry in a real-time custom-made manner (Figure 2C–E). This particular feature might contribute to the personalization of grafting procedures and a reduction of elective scan-based calculations and fitting procedures of the graft [49]. We are aware of the limitation posed by our porcine skin lesion injection model proof-of-concept further affected by artifact formation as shown in Figure 2D,E. However, we have shown the adaptability and malleability of the Osteogel, granting for future application models in bone or osteochondral lesion models.

The Osteogel emerges as a powerful in vitro osteo-conductive MSC-hydrogel interface that may also facilitate MSC pre-osteogenesis. This justifies its interpretation as a potential osteo-conductive and osteo-inductive interface beyond in vitro settings. Consequently, the Osteogel could be applied at an early time point after a bone injury and subsequently support MSC post-graft osteogenesis. All of the above qualifies the Osteogel to function as a co-injected, ready-to-use delivery device for in vivo applications of in situ bone repair, particularly as a CTEP with MSCORS.

## 4. Materials and Methods

### 4.1. MSC Cell Sources

Sampling of hair follicles was institutionally approved by the ethical committee of the Medical Faculty, University of Leipzig, Germany (427/16-ek). The human tissue was collected with written informed consents from eligible donors. Human hair follicles were plucked non-invasively from 3 healthy donors (*n* = 3, 2 sampling sessions) yielding 60 anagen hairs per session. Adipose tissue was obtained from 3 healthy donors (*n* = 3) who underwent surgery at the Department of Orthopedic, Trauma and Plastic Surgery at Leipzig University Clinic, Germany. Bone marrow mesenchymal stem cells from 3 healthy donors (*n* = 3) were provided by the Institute for Physiological Chemistry, Technical University Dresden, Germany. The cells of each donor were used between P1 and P5. Experiments were performed 3 times with 3 technical replicates, from 3 healthy donors.

### 4.2. Isolation of MSCORS, ADMSC and BMMSC

The method to obtain MSCORS from human hair follicles was described in detail in our previous research [10]. Briefly, anagen hair follicles were non-invasively plucked from donor’s occipital region. Hair shafts were shortened to 2 mm length, and a proximal part (dermal papilla) of the follicle was excised to eliminate the dermal carry-over. The shortened follicles were extensively washed in washing DPBS, and digested in 5 mg/mL collagenase X (Sigma-Aldrich GmbH, Schnelldorf, Germany) for 12 min. After neutralization and brief rinsing, hair follicles were placed onto a 0.4 μm-pore Transwell membrane (Corning Inc., New York, NY, USA) in 6 well format, and the lower chamber was filled with MSCORS isolation medium (Table 1). The follicles were cultivated in the air–liquid interface and hypoxic conditions (5% O_2_, 5% CO_2_) at 37 °C for 21 days. The cells migrated from the ORS onto the porous membrane and formed a monolayer. Upon reaching confluence, the cell layers were separated from the membrane using 0.04%/0.03% Trypsin/EDTA (PromoCell GmbH, Heidelberg, Germany) obtaining a single cell suspension, and plated onto 6 well plate in MSC Cultivation Medium (Table 1). After 24 h, the adherent cells started to proliferate, giving rise to the primary cell culture of MSCORS.

To obtain ADMSC, adipose tissue was vigorously rinsed in DPBS containing penicillin/streptomycin, chopped into 2 × 2 mm pieces and digested in 2 mg/mL Collagenase X (Sigma-Aldrich GmbH, Schnelldorf, Germany) at 37 °C for 4 h with intermittent shaking. After neutralizing with FBS, the digested tissue was intensively vortexed and centrifuged. The cell-containing pellet was washed in PBS, filtered with a 100 μm nylon strainer, and seeded into 75 cm^2^ flasks in the MSC Cultivation Medium. After 24 h, unattached cells were removed using PBS rinsing and the attached and proliferating cells were cultured as ADMSC.

BMMSC were isolated from bone marrow aspirates as described in [50]. Briefly, bone marrow aspirate was diluted in PBS and centrifuged in the Percoll solution. Mononuclear cell layer was extracted, washed in PBS, and plated into 75 cm^2^ flasks in the MSC Cultivation Medium in hypoxic conditions (5% O_2_, 5% CO_2_) at 37 °C. After 24-h attachment, unattached cells were discarded by PBS washing and attached cells were cultivated as primary BMMSC.

All MSCs were cultivated in MSC Expansion Medium until 90% confluence and subcultured at the passage ratio of 1:2 or 1:3.

### 4.3. Osteogel Fabrication, Cell Encapsulation and Differentiation in 3D Culture

The method of preparing a gelatin-based hydrogel for encapsulating MSCs was described previously [25]. Briefly, ADMSC, BMMSC, and MSCORS before P5 were grown until 90% confluent, dissociated, and re-suspended at a density of 100,000 cells/50 μL in a 5.4% (*w*/*v*) gelatin type A 300 bloom (G300) solution which was prepared in pre-warmed normal DMEM low glucose medium containing 10% Fetal Bovine Serum (FBS) and 2mM L-glutamine. Aqueous solution of triethanolamine (TEOA) was prepared as the base to maintain the crosslinking reaction. The oligomeric cross-linker (oPHpVpMA-3.5) was synthesized from pentaerythritol diacrylate monostearate (PEDAS, P), 2-hydroxypropyl acrylate (Hp), *N*-vinylpyrrolidone (Vp) in a molar ratio of 1:8.25:8.25:3.5 and dissolved in a mix of dimethyl sulfoxide (DMSO) and *N*-methyl-2-pyrrolidone (NMP) (3:1 (*v*/*v*) at a concentration of 60% (*w*/*v*). Formula and concentrations of Osteogel component in forming solutions and in stock solutions were described as follows: three compounds, gelatin cell suspension (50 μL), triethanolamine (TEOA) base (30 μL), and the oligomer solution (10 μL) were co-injected into the cap of a 500 μL Eppendorf reaction tube used as a mold and mixed by stirring. It formed a gel instantly on mixing. A total of 500 μL of DMEM containing 10% FBS was added to the 24-well plate 30 min after the gelation. After 48h, the medium was replaced by 500 μL osteogenic differentiation medium to induce osteogenic differentiation. The medium was changed twice a week in constant intervals.

### 4.4. Porcine Wound Model

A porcine ex vivo wound model was used to assess whether the semi-solid Osteogel was capable to custom-fill a wound bed with an irregular shape, solidify, and retain the acquired form. Porcine skin contains a high fraction of type 1 collagen, which is present in all tissues and with a high content in bone ECM, therefore presenting a basic yet valid model for simple ex vivo tissue analyses [51]. The skin from a porcine ear lobe was obtained from a local abattoir and cropped into smaller pieces using a 1 cm biopsy punch tool. The full-thickness wound bed was produced using a 5 mm punch on a 1 cm-size skin specimen. An amount of 300 μL Osteogel was prepared as previously described and the components were co-injected into the wound bed. After solidification, Osteogel custom-filled wound beds were cryosectioned at 5 μm thickness and stained using Hematoxylin and Eosin staining (H&E, Carl Roth GmbH, Karlsruhe, Germany).

### 4.5. Calcium Assay

Calcium deposition was quantified by means of a Calcium CPC (5+2) Assay Kit (o-cresolphthalein complexone, Greiner Diagnostic GmbH, Bahlingen, Germany). For 3D differentiation, the Osteogel was first lysed in 1 mg/mL Proteinase K (Sigma-Aldrich GmbH, Schnelldorf, Germany) in 1 N HCl solution. The supernatant of lysates containing dissolved calcium was incubated with CPC working solution, and the absorbance was measured at 575 nm with a Synergy H1 spectrophotometry plate reader (BioTek Inc., Winooski, VT, USA). Calcium concentration was calculated using the standard curve of calcium standard (10 mg/mL) and its dilutions.

### 4.6. Alkaline Phosphatase(ALP) and DNA Quantification

To quantify ALP catalytic activity in the 3D Osteogel differentiation, an ALP quantitative assay using 4-nitrophenyl phosphate (pNPP) as a substrate was used as previously described [29,52,53,54]. Briefly, differentiated cells in Osteogel were rinsed with DPBS, collected in 1.5 M Tris-HCl lysis buffer (1 mM ZnCl_2_, 1 mM MgCl_2_ and 1% Triton X-100) pH 10, and homogenized by sonication. The supernatant atop lysates was incubated with 100 μL pNPP substrate solution (3.7 mM pNPP in 100 mM diethanolamine and 0.1% Triton X-100, pH 9.8) at 37 °C for 30 min. Absorbance of the released 4-nitrophenolate was measured by a spectrophotometer at 405 nm wavelength. The overall concentration of ALP was calculated against the values of the p-nitrophenolate standard curve.

To normalize the ALP activity to the viable cell number inside the Osteogel, the DNA content was quantified using Quant-iT™ PicoGreen™ dsDNA Assay (ThermoFisher Scientific Inc., Waltham, MA, USA). Briefly, the Osteogel lysates from the previous step were added to a flat-bottom 96-well plate containing PicoGreen solution and incubated in the dark for 5 min. The fluorescence of PicoGreen was measured by a spectrophotometer set at 532 nm wavelength. The DNA concentration was calculated from the standard curve derived from the dilution of Lambda DNA standard (100 μg/mL) provided by the kit. In all samples, the average ALP was calculated from the overall ALP normalized to the DNA content of the samples, as the ALP activity per 1 μg DNA.

### 4.7. Osteogenic Gene Expression by Quantitative Real-Time Polymerase Chain Reaction (qRT-PCR)

Osteogenic gene expression was determined using qRT-PCR as previously described [10]. Briefly, differentiated MSCs in the 3D Osteogel were collected in Qiazol Lysis Reagent (Qiagen, Hilden, Germany) and homogenized using QIAGEN TissueLyser II (Qiagen, Hilden, Germany). Total RNA was isolated using RNeasy Plus Universal Kit (Qiagen, Hilden, Germany), and 1 μg of mRNA was reverse transcribed into cDNA using QuantiTect Reverse Transcription Kit (Qiagen, Hilden, Germany). Quantitative RT-PCR for targeted genes was performed using QuantiFast SYBR^®^ Green PCR Kit (Qiagen, Hilden, Germany). A total of 5–50 ng cDNA was used for each 20 μL reaction. Thermal cycling was carried out at 95 °C for 60 s, followed by 40 cycles of 95 °C for 10 s, and 60 °C for 30 s. Gene expression levels were analyzed using 7500 Software v2.3 (ThermoFisher Scientific Inc., Waltham, MA, USA), calculated using the 2^−ΔCt^ method for relative quantification, and normalized to the housekeeping gene hypoxanthine-guanine phosphoribosyltransferase (HPRT). Primers were designed using Primer3 web version 4.1.0 (60 °C annealing temperature) and manufactured by Invitrogen. The primer sequences are specified in Table 2.

### 4.8. Immunostaining of Cells within the Osteogel

To evaluate the phenotypes of the MSCs encapsulated within the Osteogel, an immunostaining of CD90 was performed. Cells were cultured in the Osteogel for 7 days, washed with DPBS, then fixed with 4% PFA and blocked with 10% Normal Goat Serum for 2 h. Primary anti-human CD90 antibody (Clone 5E10; Sigma-Aldrich GmbH, Schnelldorf, Germany; 1:100 dilution) was added to the cells overnight at 4 °C. Washed using PBS containing 0.5% Tween 20, Osteogels were incubated with secondary AlexaFluor^®^ 594-conjugated goat anti-mouse IgG antibody (ThermoFisher Inc., Waltham, MA, USA; 1.0 mg/mL, 1:200 dilution) and 4′,6-diamidino-2-phenylindole DAPI (ThermoFisher Inc., Waltham, MA, USA; 1:400 dilution). After intensive washing, the immunofluorescent staining was documented by Keyence BZ-9000 Fluorescence Microscope (Keyence GmbH, Neu-Isenburg, Germany).

### 4.9. Live/Dead Cell Assay

To determine cell viability and cytotoxicity of MSCs in Osteogel in 3D osteo-differentiation conditions, a Live/Dead Assay (ThermoFisher Inc., Waltham, MA, USA) consisting of Calcein AM and Propidium Iodide (PI) was used. Briefly, Osteogel containing the cells was washed with DPBS, and stained with Calcein AM (1:400) and PI (1:200) for 15 min. The fluorescent signals were captured by z-stacking at multiple focused planes shifted by 5 µm and reconstructed in 3D using Zeiss LSM 700 confocal microscope (Carl Zeiss AG, Oberkochen, Germany).

### 4.10. Statistical Analysis

All quantitative results were statistically analyzed using an unpaired t-test or nonparametric Mann–Whitney test. Normal distribution and homogeneity of variance of datasets were checked using a Shapiro–Wilk normality test and F-test. *p* Values ≤ 0.05 were considered statistically significant (* *p* < 0.05, ** *p* < 0.01, *** *p* < 0.005).

## 5. Conclusions

In conclusion, ADMSCs, BMMSCs, and MSCORS achieved a consistent differentiation towards the osteogenic lineage in our proposed 3DOsteogel model, retaining high cell viability, good expression levels of key osteogenic markers, functional ALP, and substantial CaP deposition. MSCORS displayed a particularly high osteo-differentiation potential in the Osteogel, even superseding that of ADMSCs and BMMSCs in terms of mineral deposition and expression of OCN and BMP2, key osteogenic marker genes. Our findings suggest that we have identified a good model of aECM with osteo-differentiative/supportive properties, when combined with various MSCs, which could serve as a delivery device in further investigation of bone grafting models. Moreover, the non-invasive harvesting of the MSCORS, together with their high capacity for differentiation and personalization could help with further developing the Osteogel into a novel CTEP, optimally fitting a philosophy of ‘bench-to-bedside’ application.

## Figures and Tables

**Table 1:** Medium compositions for cell culture.

Medium	Medium Composition
MSCORS Washing Medium	DMEM (Low Glucose) 100 U/mL Penicillin 100 μg/mL Streptomycin 50 μg/mL Gentamycin 10 μg/mL Amphotericin B
MSCORS Isolation Medium	DMEM (Low Glucose) 10% Fetal Bovine Serum 1% ITS Premix 10 ng/mL bFGF 20 ng/mL rhEGF 2 mM L-Glutamine 1% Pen/Strep (Penicillin 100 U/mL, Streptomycin 100 μg/mL)
MSCORS/ADMSC Culture Medium	DMEM (Low Glucose) 10% Fetal Bovine Serum 2 mM L-Glutamine 1% Pen/Strep (Penicillin 100 U/mL, Streptomycin 100 μg/mL)
MSC Osteogenic Medium	DMEM (Low Glucose) 10% Fetal Bovine Serum 2 mM L-Glutamine 200 nM Dexamethasone 50 ug/mL Ascorbic Acid 10 mM β-glycerophosphate

**Table 2:** **qRT-PCR** Primer sequences.

Gene		Primer Sequence
Runx2	for	AATGACACCACCAGGCCAAT
Runx2	rev	TGGCCTACAAAGGTGGGTTT
TNAP	for	AGAACCCCAAAGGCTTCTTC
TNAP	rev	CTTGGCTTTTCCTTCATGGT
OCN	for	GGTGCAGCCTTTGTGTCCAAGC
OCN	rev	GTCAGCCAACTCGTCACAGTCC
BMP2	for	GGCATCCTCTCCACAAAAGA
BMP2	rev	GTGGCAGTAAAAGGCGTGAT
PPARG	for	TACTGTCGGTTTCAGAAATGCC
PPARG	rev	GTCAGCGGACTCTGGATTCAG
HTRP-1	for	ACCACCGTGTGTTAGAAAAGT
HTRP-1	rev	CTGCTGACAAAGATTCACTGGT

## Data Availability

The data presented in this study are available on request from the corresponding author.

## References

[1] Campana V., Milano G., Pagano E., Barba M., Cicione C., Salonna G., Lattanzi W., Logroscino G. (2014). Bone substitutes in orthopaedic surgery: From basic science to clinical practice. J. Mater. Sci. Mater. Med..

[2] de Grado G.F., Keller L., Idoux-Gillet Y., Wagner Q., Musset A.M., Benkirane-Jessel N., Bornert F., Offner D. (2018). Bone substitutes: A review of their characteristics, clinical use, and perspectives for large bone defects management. J. Tissue Eng..

[3] Caplan A.I. (2007). Adult mesenchymal stem cells for tissue engineering versus regenerative medicine. J. Cell. Physiol..

[4] Samsonraj R.M., Raghunath M., Nurcombe V., Hui J.H., van Wijnen A.J., Cool S.M. (2017). Concise Review: Multifaceted Characterization of Human Mesenchymal Stem Cells for Use in Regenerative Medicine. Stem Cells Transl. Med..

[5] Sheng G. (2015). The developmental basis of mesenchymal stem/stromal cells (MSCs). BMC Dev. Biol..

[6] Haynesworth S., Goshima J., Goldberg V., Caplan A. (1992). Characterization of cells with osteogenic potential from human marrow. Bone.

[7] Zuk P.A., Zhu M., Ashjian P., de Ugarte D.A., Huang J.I., Mizuno H., Alfonso Z.C., Fraser J.K., Benhaim P., Hedrick M.H. (2002). Human adipose tissue is a source of multipotent stem cells. Mol. Biol. Cell.

[8] Fraser J.K., Wulur I., Alfonso Z., Hedrick M.H. (2006). Fat tissue: An underappreciated source of stem cells for biotechnology. Trends Biotechnol..

[9] Anker P.S.i., Scherjon S.A., der Keur C.K., de Groot-Swings G.M., Claas F.H., Fibbe W.E., Kanhai H.H. (2004). Isolation of mesenchymal stem cells of fetal or maternal origin from human placenta. Stem Cells.

[10] Li H., Masieri F.F., Schneider M., Kottek T., Hahnel S., Yamauchi K., Obradović D., Seon J.-K., Yun S.J., Ferrer R.A. (2020). Autologous, Non-Invasively Available Mesenchymal Stem Cells from the Outer Root Sheath of Hair Follicle Are Obtainable by Migration from Plucked Hair Follicles and Expandable in Scalable Amounts. Cells.

[11] Bertram J.E., Polevoy Y., Cullinane D.M. (1998). Mechanics of avian fibrous periosteum: Tensile and adhesion properties during growth. Bone.

[12] Cho D.I., Kim M.R., Jeong H.Y., Jeong H.C., Jeong M.H., Yoon S.H., Kim Y.S., Ahn Y. (2014). Mesenchymal stem cells reciprocally regulate the M1/M2 balance in mouse bone marrow-derived macrophages. Exp. Mol. Med..

[13] Romieu-Mourez R., François M., Boivin M.N., Bouchentouf M., Spaner D.E., Galipeau J. (2009). Cytokine modulation of TLR expression and activation in mesenchymal stromal cells leads to a proinflammatory phenotype. J. Immunol..

[14] Asami T., Ishii M., Fujii H. (2013). Macrophage TLR7/8-Mediated Cytokine Expression by Mesenchymal Stem Cell-Conditioned Medium. Mediat. Inflamm..

[15] Ciuffreda M.C., Malpasso G., Musarò P., Turco V., Gnecchi M. (2016). Protocols for in vitro Differentiation of Human Mesenchymal Stem Cells into Osteogenic, Chondrogenic and Adipogenic Lineages. Methods Mol. Biol..

[16] Amini A.R., Laurencin C.T., Nukavarapu S.P. (2012). Bone tissue engineering: Recent advances and challenges. Crit. Rev. Biomed. Eng..

[17] Zhang T., Lin S., Shao X., Zhang Q., Xue C., Zhang S., Lin Y., Zhu B., Cai X. (2017). Effect of matrix stiffness on osteoblast functionalization. Cell Prolif..

[18] Engler A.J., Sen S., Sweeney H.L., Discher D.E. (2006). Matrix elasticity directs stem cell lineage specification. Cell.

[19] Park J.S., Chu J.S., Tsou A.D., Diop R., Tang Z., Wang A., Li S. (2011). The effect of matrix stiffness on the differentiation of mesenchymal stem cells in response to TGF-beta. Biomaterials.

[20] Loth T., Hötzel R., Kascholke C., Anderegg U., Schulz-Siegmund M., Hacker M.C. (2014). Gelatin-based biomaterial engineering with anhydride-containing oligomeric cross-linkers. Biomacromolecules.

[21] Loth T., Hennig R., Kascholke C., Hötzel R., Hacker M.C. (2013). Reactive and stimuli-responsive maleic anhydride containing macromers–multi-functional cross-linkers and building blocks for hydrogel fabrication. React. Funct. Polym..

[22] Hosaka A., Koyama H., Kushibiki T., Tabata Y., Nishiyama N., Miyata T., Shigematsu H., Takato T., Nagawa H. (2004). Gelatin Hydrogel Microspheres Enable Pinpoint Delivery of Basic Fibroblast Growth Factor for the Development of Functional Collateral Vessels. Circulation.

[23] Chong E.J., Phan T.T., Lim I.J., Zhang Y.Z., Bay B.H., Ramakrishna S., Lim C.T. (2007). Evaluation of electrospun PCL/gelatin nanofibrous scaffold for wound healing and layered dermal reconstitution. Acta Biomater..

[24] Echave M.C., del Burgo L.S., Pedraz J.L., Orive G. (2017). Gelatin as Biomaterial for Tissue Engineering. Curr. Pharm. Des..

[25] Nawaz H.A., Schröck K., Schmid M., Krieghoff J., Maqsood I., Kascholke C., Kohn-Polster C., Schulz-Siegmund M., Hacker M.C. (2021). Injectable oligomer-cross-linked gelatine hydrogels via anhydride–amine-conjugation. J. Mater. Chem. B.

[26] Li H., Masieri F.F., Schneider M., Bartella A., Gaus S., Hahnel S., Zimmerer R., Sack U., Maksimovic-Ivanic D., Mijatovic S. (2021). The Middle Part of the Plucked Hair Follicle Outer Root Sheath Is Identified as an Area Rich in Lineage-Specific Stem Cell Markers. Biomolecules.

[27] Savkovic V., Li H., Obradovic D., Masieri F.F., Bartella A.K., Zimmerer R., Simon J.C., Etz C., Lethaus B. (2021). The Angiogenic Potential of Mesenchymal Stem Cells from the Hair Follicle Outer Root Sheath. J. Clin. Med..

[28] Dominici M., le Blanc K., Mueller I., Slaper-Cortenbach I., Marini F., Krause D., Deans R., Keating A., Prockop D., Horwitz E. (2006). Minimal criteria for defining multipotent mesenchymal stromal cells. Int Soc. Cell. Ther. Position Stat. J. Cytother..

[29] Vogel S., Arnoldini S., Möller S., Schnabelrauch M., Hempel U. (2016). Sulfated hyaluronan alters fibronectin matrix assembly and promotes osteogenic differentiation of human bone marrow stromal cells. Sci. Rep..

[30] Pittenger M.F., Mackay A.M., Beck S.C., Jaiswal R.K., Douglas R., Mosca J.D., Moorman M.A., Simonetti D.W., Craig S., Marshak D.R. (1999). Multilineage potential of adult human mesenchymal stem cells. Science.

[31] Conget P.A., Minguell J.J. (1999). Phenotypical and functional properties of human bone marrow mesenchymal progenitor cells. J. Cell. Physiol..

[32] Haynesworth S., Barer M., Caplan A. (1992). Cell surface antigens on human marrow-derived mesenchymal cells are detected by monoclonal antibodies. Bone.

[33] le Blanc K., Tammik C., Rosendahl K., Zetterberg E., Ringdén O. (2003). HLA expression and immunologic propertiesof differentiated and undifferentiated mesenchymal stem cells. Exp. Hematol..

[34] Baksh D., Yao R., Tuan R.S. (2007). Comparison of proliferative and multilineage differentiation potential of human mesenchymal stem cells derived from umbilical cord and bone marrow. Stem Cells.

[35] Batsali A., Pontikoglou C., Kouvidi E., Damianaki A., Stratigi A., Kastrinaki M.-C., Papadaki H.A. (2013). Comparative Analysis of Bone Marrow and Wharton’s Jelly Mesenchymal Stem/Stromal Cells. Blood.

[36] Bidarra S.J., Barrias C.C., Barbosa M.A., Soares R., Amedee J., Granja P.L. (2011). Phenotypic and proliferative modulation of human mesenchymal stem cells via crosstalk with endothelial cells. Stem Cell Res..

[37] Xiao G., Gopalakrishnan R., Jiang D., Reith E., Benson M.D., Franceschi R.T. (2002). Bone morphogenetic proteins, extracellular matrix, and mitogen-activated protein kinase signaling pathways are required for osteoblast-specific gene expression and differentiation in MC3T3-E1 cells. J. Bone Min. Res..

[38] Huang W., Yang S., Shao J., Li Y.P. (2007). Signaling and transcriptional regulation in osteoblast commitment and differentiation. Front. Biosci..

[39] An Q., Wu D., Ma Y., Zhou B., Liu Q. (2015). Suppression of Evi1 promotes the osteogenic differentiation and inhibits the adipogenic differentiation of bone marrow-derived mesenchymal stem cells in vitro. Int. J. Mol. Med..

[40] Phillips J.E., Gersbach C.A., Wojtowicz A.M., Garcia A.J. (2006). Glucocorticoid-induced osteogenesis is negatively regulated by Runx2/Cbfa1 serine phosphorylation. J. Cell Sci..

[41] Ding H., Chen S., Yin J.H., Xie X.T., Zhu Z.H., Gao Y.S., Zhang C.Q. (2014). Continuous hypoxia regulates the osteogenic potential of mesenchymal stem cells in a time-dependent manner. Mol. Med. Rep..

[42] Rowlands A.S., George P.A., Cooper-White J.J. (2008). Directing osteogenic and myogenic differentiation of MSCs: Interplay of stiffness and adhesive ligand presentation. Am. J. Physiol.-Cell Ph..

[43] Evers E.E., Zondag G.C., Malliri A., Price L.S., Klooster J.P.T., van der Kammen R.A., Collard J.G. (2000). Rho family proteins in cell adhesion and cell migration. Eur. J. Cancer.

[44] Maharam E., Yaport M., Villanueva N.L., Akinyibi T., Laudier D., He Z., Leong D.J., Sun H.B. (2015). Rho/Rock signal transduction pathway is required for MSC tenogenic differentiation. Bone Res..

[45] Yamamoto T., Ugawa Y., Yamashiro K., Shimoe M., Tomikawa K., Hongo S., Kochi S., Ideguchi H., Maeda H., Takashiba S. (2014). Osteogenic differentiation regulated by Rho-kinase in periodontal ligament cells. Differentiation.

[46] Sun M., Chi G., Li P., Lv S., Xu J., Xu Z., Xia Y., Tan Y., Xu J., Li L. (2018). Effects of Matrix Stiffness on the Morphology, Adhesion, Proliferation and Osteogenic Differentiation of Mesenchymal Stem Cells. Int. J. Med. Sci..

[47] da Violante G., Zerrouk N., Richard I., Provot G., Chaumeil J.C., Arnaud P. (2002). Evaluation of the cytotoxicity effect of dimethyl sulfoxide (DMSO) on Caco2/TC7 colon tumor cell cultures. Biol. Pharm. Bull..

[48] Still P.C., Yi B., González-Cestari T.F., Pan L., Pavlovicz R.E., Chai H.B., Ninh T.N., Li C., Soejarto D.D., McKay D.B. (2013). Alkaloids from Microcos paniculata with cytotoxic and nicotinic receptor antagonistic activities. J. Nat. Prod..

[49] Maisani M., Pezzoli D., Chassande O., Mantovani D. (2017). Cellularizing hydrogel-based scaffolds to repair bone tissue: How to create a physiologically relevant micro-environment?. J. Tissue Eng..

[50] Oswald J., Boxberger S., Jørgensen B., Feldmann S., Ehninger G., Bornhäuser M., Werner C. (2004). Mesenchymal Stem Cells Can Be Differentiated Into Endothelial Cells In Vitro. Stem Cells.

[51] Jacobi U., Kaiser M., Toll R., Mangelsdorf S., Audring H., Otberg N., Sterry W., Lademann J. (2007). Porcine ear skin: An in vitro model for human skin. Ski. Res. Technol..

[52] Hempel U., Hefti T., Kalbacova M., Wolf-Brandstetter C., Dieter P., Schlottig F. (2010). Response of osteoblast-like SAOS-2 cells to zirconia ceramics with different surface topographies. Clin. Oral Implant. Res..

[53] Hempel U., Möller S., Noack C., Hintze V., Scharnweber D., Schnabelrauch M., Dieter. P. (2012). Sulfated hyaluronan/collagen I matrices enhance the osteogenic differentiation of human mesenchymal stromal cells in vitro even in the absence of dexamethasone. Acta Biomater..

[54] Hempel U., Preissler C., Vogel S., Möller S., Hintze V., Becher J., Schnabelrauch M., Rauner M., Hofbauer L.C., Dieter P. (2014). Artificial extracellular matrices with oversulfated glycosaminoglycan derivatives promote the differentiation of osteoblast-precursor cells and premature osteoblasts. BioMed Res. Int..

